# Attitudes and opinions of Oral healthcare professionals on screening for Type-2 diabetes

**DOI:** 10.1186/s12913-021-06756-y

**Published:** 2021-07-27

**Authors:** Rodrigo Mariño, Andre Priede, Michelle King, Geoffrey G. Adams, Diego Lopez

**Affiliations:** Melbourne Dental School, University of Melbourne, Melbourne, Australia

**Keywords:** Oral health, Type 2 diabetes, KAPB, Oral health professionals

## Abstract

**Background:**

As part of a larger study on the identification of undiagnosed Type 2 diabetes (T2D), and prediabetes patients in dental settings, this study explored oral healthcare professionals’ (OHP) attitudes with respect to the relevance and appropriateness of screening for prediabetes/T2D in general oral healthcare settings. It also aims to gain a deeper understanding of OHPs’ concerns and perceived barriers to screening for T2D.

**Methods:**

Semi-structured interviews were conducted with 11 OHPs: eight dentists, two dental hygienists and one oral health therapist. Interviews were audio recorded, transcribed verbatim and analysed using thematic analysis.

**Results:**

Themes that emerged from the interviews were organised under three major categories: 1) Implementation: OHPs willingness to screen for prediabetes/T2D; 2) Barriers to implementation of screenings; subdivided into: a) lack of knowledge and formal training about T2D screening methodology; b) concerns about patients’ awareness and acceptance of T2D screening in oral healthcare settings; c) costs and reimbursement for the time and resources required to screen patients; and d) legal and scope of practice*;* and 3) Collaboration and communication between OHPs and General practitioners (GP).

**Conclusions:**

The oral healthcare setting was considered as appropriate for medical screening, and OHPs were willing to participate in screening for prediabetes/T2D. Nonetheless, for the successful implementation of a screening programme, several barriers need to be addressed, and effective medical screening would require collaboration between oral health and medical and other health professionals, as well as clarification of legal and reimbursement issues.

## Background

Type 2 diabetes (T2D) is the most common form of diabetes, occurring in about 90% of people with diabetes [1]. T2D occurs when the body becomes resistant to the insulin being produced by the pancreas and the amount produced is inadequate to meet the body’s needs. It is is associated with a myriad of complications which affect the feet, eyes, mouth, kidneys, and cardiovascular health. T2D was the sixth leading causes of death in Australia in 2011 [2]. This type of diabetes is typically diagnosed after the age of 40, although recently T2D has also been diagnosed in younger adults and occasionally adolescents [1].

In Australia, there were over 1.2 million people (4.4% of the total population) registered with T2D in the National Diabetes Services Scheme in 2020 [1, 3]. By the year 2025, it has been estimated that up to 3 million Australians over the age of 25 years will have the condition [4].

Furthermore, it has been estimated that for every four adults with diagnosed diabetes, there would be one who was undiagnosed [5, 6]. People with undiagnosed diabetes often do not seek help until they have developed complications [2], which may occur many years after their disease began.

A related condition is prediabetes, where blood glucose levels are higher than normal but not high enough to be diagnosed as T2D. It affects nearly 1 in 6 adults (i.e., more than 2 million Australians) over the age of 25 years, who have a higher risk of developing T2D and increased risk of cardiovascular disease [7].

The high prevalence of undiagnosed diabetes, and the increased risk of diabetes and cardiovascular disease in those with pre-diabetes [7], highlights the importance of early detection of the disease. Although diabetes was declared a National Health Priority in 1997, its increasing prevalence demonstrates that still more needs to be done [8].

To address the challenges imposed by T2D, early diagnosis and treatment of T2D and prediabetes is critical to improving health outcomes. For that purpose, a coordinated, multicomponent intervention can make a significant difference. In that intervention, Governments, healthcare providers, people with diabetes and their carers, food producers, pharmaceutical companies, and the society at large would all have a role [9].

Oral health professionals (OHPs) could play a significant role in this multi-professional team. The use of OHPs (dentists, dental hygienists, dental therapists, and oral health therapists) to identify prediabetes and undiagnosed T2D has been trialled in a number of countries including the United States [10–13], Greece [14] Saudi Arabia [15] Italy [16], Denmark [17], and Nigeria [18]. This screening approach has been found to be generally well-received and accepted by both practitioners and patients [11] and these studies have been able to identify undiagnosed cases of T2D or prediabetes [12]. Although this mode of screening proved acceptable to the dentists, doctors and patients involved, for screening to be routinely adopted and effective, it must gain wider acceptance amongst OHPs. Their acceptance is critical for the successful implementation of medical screening in the oral healthcare setting.

Thus, to effectively promote chairside health screening in oral healthcare settings, it is important to understand OHPs’ views about the relevance and appropriateness of a proposed T2D screening protocol. As part of a larger study on the identification of prediabetes and T2D, this study aimed to explore OHPs’ attitudes about the relevance and appropriateness of screening for prediabetes/T2D in oral healthcare settings It also aims to gain a deeper understanding of OHPs’ concerns and perceived barriers to screening for T2D in general oral healthcare practice settings.

## Methods

Following ethical approval from the University of Melbourne Human Ethics Advisory Group (ethics ID. 1,647,537), a study using semi-structured interviews was organised to explore participants’ perceptions with regards to the format, relevance and appropriateness of the proposed approach to screening of T2D. Interviews were conducted between March and July 2018, on a one-on-one basis by one interviewer with over 10 years trials experiences including conducting qualitative interviews (MK) at a mutually convenient place or by telephone. Participants were recruited from OHPs who volunteered to be interviewed by providing contact details to an online quantitative questionnaire [19]. Eleven oral healthcare professionals: two dental hygienists (DH), one oral health therapist (OHT), and eight general dental practitioners (GDP), covering both rural and urban areas of the State of Victoria, participated in this study. Following the analysis of the 11 interviews, the research team judged that data saturation had been achieved. The interviews started by securing informed consent from each participant to participate and for the interviews to be audio recorded. Mean interview time was 28.7 min (s.d. 4.1 min). The semi-structured interviews were conducted following a topic guide consisting of four main themes related to the domains of T2D knowledge, attitudes, and practices around treating patients with suspected or confirmed T2D and prediabetes. The schedule of questions, consisting of eight questions grouped into four themes, is presented in Table 1. Each question had a list of potential probes to further explore their responses. One pilot interview was undertaken to ensure the method was appropriate to address the research questions. Interviews were transcribed verbatim using Go Transcribe [20]. Transcriptions were imported into QSR NVivo release 1.3 (535) 12.1.1 software and analysed using thematic analysis to identify key patterns, trends in the data and recurring themes.

**Table 1:** Topics discussed in qualitative interviews

Theme 1. The role of the OHP in diabetes screening.	
• Do you think oral health professionals should screen routinely for pre- Diabetes Mellitus (DM) /Diabetes Mellitus (DM)?	
Theme 2. OHP experience and current practice in diabetes screening and management.	
• What have been your experience in assessing pre-DM/DM among your patients?	
Theme 3. Education and training.	
• What training have you had?	
• Do you feel you need additional training to assess patients for pre-DM/DM?	
• How can health promotional and educational strategies establish and clarify links between pre-DM/DM and oral health?	
Theme 4. Opinions and suggested improvements	
• What do you think patients expect from oral health professionals in regard to Diabetes screening?	
• Can you suggest ways to improve assessment of pre-DM/DM in the dental clinic?	
• Do oral healthcare providers adequately address issues with pre DM/DM?.	

An initial coding of the data was carried out by systematic reading through the entire data set and familiarising with the interviews. Initial coding as free nodes resulted in 12 nodes. The themes were coded by one investigator (DL) and checked by another (RM) to provide an independent perspective on findings [21]. The reviewers’ agreement was deemed substantial (k-statistic = 0.69). Following deliberations, minor modifications were made to the coding of the data and categorisation under various themes.

Repetitive revisiting of the transcripts and audio recordings helped to collapse the nodes into broader themes. The data was analysed using thematic analysis to explore and understand the important influences on the individual perceptions. Further higher-order categories were constructed to outline interrelations between these influences [22]. Themes were data driven and were categorised based on questions asked in the interviews. An iterative process of categorising and re-categorising was involved in the thematic analysis.

## Results

The characteristics of the participant OHPs are described in Table 2. Themes that emerged from the responses to the interviews were organised under three major categories: 1) Implementation: OHPs were willing to implement screenings for prediabetes/T2D; 2) Barriers to the implementation of screening; and 3) Collaboration and communication between OHPs and General Practitioner (GP).

**Table 2:** Oral Health Professionals interviewed for study

Sex	Training/Qualifications	Number of years since graduation	Practice Descriptor Urban/Rural
Female	Dentist	23	Urban
Male	Dentist	29	Urban
Male	Dentist	23	Urban
Female	Dentist	12	Urban
Female	Dentist	38	Urban
Male	Dentist	24	Urban
Female	Dentist	27	Urban
Female	Oral Health Therapist	10	Rural
Female	Dentist	28	Urban
Female	Dental Hygienist	6	Rural
Female	Dental Hygienist	8	Urban

### Implementation: OHPs were willing to implement screenings for prediabetes/T2D

Most OHPs agreed that T2D is an issue that is getting worse globally, that OHPs are in a prime position to screen for diabetes, and they should get involved. The OHPs believed screening is an important procedure which could be beneficial to their patient’s health.

*“I think for me personally, I think it is very important because we see the patients a lot more regularly than they see their doctors. So, there are some simple things that we could very easily screen for in our surgery” (OHP 3)*

OHPs were aware of the link between diabetes and oral health and acknowledged the relevance of the oral health profession to early detection of T2D. Some OHPs even reported that as a part of providing comprehensive care to their patients they were already screening for other health conditions such as skin cancer.

Participants believed that T2D screening should be part of their regular oral healthcare practice. There was also agreement that OHPs should not delegate the screening to their clinic staff. Although, OHPs were very supportive of screening, they reflected out that after the initial conversation about T2D awareness with the patients often there was no follow-up.

*“The problem always is that often you will have initial conversations with somebody, and it all sounds really exciting and then everybody gets busy with their own lives and work and then you know sometimes things do not follow through beyond that” (OHP 5)*

It was acknowledged that it is always hard to initiate the implementation and get people to make the transition. It was suggested that to overcome this issue of follow-up and to initiate implementation, there is a need for leadership during the T2D screening implementation. In fact, some participants noted that there were practices which have already adopted a referral system to check patient follow-ups after the initial T2D screening or conversation.

“*We have a system that we follow them up six weeks later and we just see how they went, and we also double check that they wanted to go, that they did go and if they didn't want to go then that's absolutely fine, it is just all documented in our records*” *(OHP 1)*

OHPs reported that they usually identified patient with a confirmed T2D diagnosis, rather than conducting a screening. Once the patient’s diabetes status was known, it made discussions easier.

*“Well, if they are already diabetic it is easy, I just ask for their numbers and how long, particularly, how they have been diagnosed is critical factor, mainly because diagnosis is usually pretty late, and they have usually been diabetic twice that time” (OHP 2)*

T2D screening is not something that would be done routinely. The impetus for a T2D check or screening might be a patient presenting with poor periodontal health, or when a patient mentioned T2D symptom.

*“When the patient comes in and they have got some sort of uncontrolled periodontal disease or we are starting to see periodontal disease going downhill or with comments like numb toes and fingers or something like that and they have not seen a doctor, that's usually when we refer them off to a GP to get tested for diabetes” (OHP 3)*

Participants requested for a simple test for diabetes screening that can be easily incorporated in oral healthcare settings. Current tests were seen as invasive, unlike a smoking cessation intervention, for example. On the other hand, there were dentists who indicated that screening was part of a dental hygienists’ duties, or of those who run the first general check-up with that patient.

Interestingly, one OHP highlighted that dental caries is not mentioned in other health professions’ research, apart from the relationship with sugar.

*“The research […] do not even mentioned dental decay, aside mentioning sugar and all sorts of things” (OHP 2)*

Thus, for successful implementation, more training is needed, not only for OHPs, but also for GPs, nutritionists, and diabetes educators. Continuing Professional Development (CPD) should be available for all health professionals.

*“Education, I think, needs to go both ways as well. So, there is an awareness from diabetes educators about the importance of oral health and that you know we are all working together with similar messages. And cross-referring in both directions, not just in one direction*” *(OHP 5)*

### Barriers exist to the implementation of screening

This theme included four sub-themes: a) lack of knowledge and formal training about T2D screening methodology; b) concerns about patients’ awareness and acceptance of T2D screening in oral healthcare settings; c) costs and reimbursement for the time and resources required to screen patients; and d) legal and scope of practice*.*

#### Lack of knowledge and formal training about T2D screening methodology

Some of the OHPs mentioned that there was a lack of knowledge and training on how to conduct more in-depth screenings on their patients as they felt that they might not be able to respond to all the patient’s questions.

*“I mean there is really no training in dentistry at all to allow dentists to regularly screen or council for diabetes so that's traditional” (OHP 2)*

For some OHPs, T2D was not part of the dental curriculum at University, while other OHPs recalled that it was, but said that was the last time they were exposed to this topic. As such, OHPs indicated that it was an area where there was a need for training.

*“You know to be honest; diabetes screening was not really part of our training. It is really, maybe we were only told, like at university I think we were only told to maybe discuss it. But I do not know, we didn't do any sort of diabetes screening as part of our training” (OHP 8)*

While graduate dental courses now cover the relationship between oral health and systemic health including diabetes, OHPs indicated that there were few CPD courses available that provide an update on this topic.

Another attitudinal barrier mentioned by some OHPs is that screening for T2D or any chronic conditions is not their responsibility.

*“I would not say that I feel it is our responsibility to do it. I think it would be going above our responsibility*.” *(OHP 10)*

Furthermore, even when OHPs believed that was part of their duty of care, they were reluctant to take blood samples or finger prick tests for diabetes screening:

*“So as far as definitive screening if that's involving a blood test I think that's more for the GP” (OHP 6)*

#### Concerns about patients’ awareness and acceptance of T2D screening in oral healthcare settings

OHPs believed patients are usually unaware of the link between oral health and general health and consequently do not expect to receive T2D screenings during consultation:

*“I do not think people understand the connection, so I think making people aware of the connection”. (OHP 1)*

“*Oh, you're not my doctor why are you talking about this to me*” *(OHP 2)*

To improve awareness, participants also mentioned the need to advise patients and diabetes educators about the bidirectional relationship between oral health and diabetes [23].

“*Most of my patients who are confirmed diabetics and see diabetes educators have no idea that it could affect their teeth or vice versa*” *(OHP 3)*

Despite a lack of patient awareness about T2D and its association with oral health being reported as a barrier for implementing screenings in oral healthcare practices, some patients were enthusiastic when offered a T2D screening. In particular, after the importance and purpose of screening was explained. In fact, participants mentioned a few cases when patients were grateful for the screening, as their prediabetes was detected before it developed into T2D.

*“I send them off to their doctors and then they'll go and do a glucose tolerance test and then it all comes back to me six months later saying thank you so much for that I was able to get on top of my prediabetic state and able to control it before it went into full blown type 2” (OHP 1)*

It was also reported that there is a general feeling that despite barriers and initial resistance, patients would accept the screening.

“*I can say overwhelmingly appreciative. I can say they feel like you are going above and beyond, and it is all in the way that you present it to them. Obviously, you do not want to scare them*” *(OHP 1)*

Still for some, OHP preferred to recommend a visit to a GP. Participants indicated that this was due to two main issues. Firstly, the practicalities of the screening and assessment. For example, waist measurement is part of the Australian type 2 diabetes risk assessment tool (AUSDRISK) and patients may not expect to measure their waist in the waiting room [24]. Secondly, there are so many things to be done at the same time and within the appointment time.

#### Costs and reimbursement, time and resources required to screen patients

Participants considered that T2D screenings should be implemented in both private and public practices. However, in Australia, screening T2D in oral healthcare settings is not part of Australia’s universal health insurance scheme (Medicare). There is no Australian Dental Association’s code for general health screenings, and private health insurance companies do not reimburse the patient for them. Therefore, there is no compensation for the time invested in these screenings. Those who do screenings included it as part of the general examination costs, but for it to become a common practice there is a need to persuade insurance companies and the Australian Dental Association for a code to charge for it.

*“I am guessing that there might be resources we require. So, if we are going to be doing the blood prick tests or whatever we would be needing that equipment and in order to have that equipment that's the practice I guess, you know, no one is just going to go and buy that if the government or insurers are not helping cover the cost yet” (OHP 8)*

Due to this lack of reimbursement for the time invested in screenings, there is no economic incentive to conduct them in oral healthcare settings. Consequently, OHPs employed in large organisations, may not be encouraged to promote this practice, even though they may feel T2D screening is beneficial for the public health. This was particularly the case for OHPs working in group clinics, where the clinicians are employees, and have performance goals to achieve.

” *Obviously if my bosses were supporting it, that would allow the time and provide the money to do it…” (OHP 11)*

#### Legal and scope of practice

Ethical and legal issues were also mentioned as a potential barrier to diabetes screening in the dental setting. OHPs, including dentist, were uncertain as to whether diabetes screening was within their scope of practice.

“*I mean it is also about the scope of practice because certainly in terms of our registration as dentists there needs to be evidence that we're working within our scope and so there would need to be some discussion about whether doing blood tests for diabetes was seen as something within our scope of practice*” *(OHP 5)*

Additionally, OHPs reported some concern with screening using biochemical tests (i.e**.,** urine glucose, blood glucose, HbA1c).

*“It depends on the type of blood test you do. In drawing blood, I do not feel comfortable with. But if it were a skin prick test, I wouldn't have any issues of that” (OHP 3)*

On the other hand, some participants indicated that preforming a random capillary blood glucose measurement would be trivial compared to other activities done in dentistry.

“*I think in terms of, you know, pricking people's fingers I mean it is trivial compared to giving dental injection so that's not a problem. So, I think the mechanics of it are not the problem*” *(OHP 2)*

### Collaboration and communication between OHPs and GPs

The need for referral pathways was cited as fundamental for the introduction of T2D screenings in routine practice.

“*I see barriers with the medical profession because certainly in the dental profession we are currently going through a situation where we are talking about other health professionals doing our jobs as well. So just see it as blurring the lines as long as the GP is on side, and they are happy with you doing that. I think that is a courtesy issue” (OHP 6)*

“*So that’s always a tricky one because for some reason dentistry always seems to be off on one side and is it historically been quite difficult to integrate dentistry into general health settings*” *(OHP 5)*

There is also a need to improve communication and referral pathways to GPs for medical follow-up, to ensure the diabetes screening protocol is completed. Nonetheless, some interviewees did not have a clear idea of how to refer patients to GPs.

In this study, OHPs gave very positive feedback about the suitability and acceptability of T2D screening and referral pathways (iDENTify) [25]. Nonetheless, the outcome of the medical follow-up was usually reported to the OHP by the patient and not a formal communication from the GP.

“*So, I find quite often without a letter you just get a verbal response, and you know you can follow up with the patience but difficult to get on to the GP.” (OHP 10)*

## Conclusions

OHPs are in a unique position to screen, or facilitate the diagnosis of many chronic conditions, not just conditions of the oral cavity. Our findings indicate that, although OHPs view screening for T2D as part of their role and it is important to the overall health of their patients, screening for T2D was not done routinely. Consequently, OHPs would not routinely identify patients in early stage T2D or prediabetes. Screening and referral may occur after the medical history is taken, or because the patient presents with overt symptoms, but this is not considered screening, but diagnosis [26]. The main purpose of screening is to detect asymptomatic individuals at a high-risk of having prediabetes or T2D. ‘Opportunistic’ screening of patients at high risk of T2D can be done using simple screening methods such as the AUSDRISK tool.

Consistent with previous literature [27], time and cost were considered the most significant barriers to implementing screening for T2D in the oral healthcare settings in this study. Participants also recognised that they may not have the required knowledge and training and may need additional refresher courses on the topic, prior to implementing diabetes screening. In addition, if T2D screening is going to become part of their routine practice, there is a need for clarification of the legal framework and scope of practice associated with the diabetes screening modality chosen. For example, if a blood glucose measurement is to be used, it requires an invasive procedure, and the Australian Diabetes Educator Association recommends that measuring capillary blood glucose levels using blood glucose meter should be limited to individuals who have received training in the use of the blood glucose meter and testing strips and are subsequently able to demonstrate competency in their use [26]. On the other hand, OHPs’ education and training should enable them to competently implement a survey based, non-invasive risk assessment tool such as the AUSDRISK and therefore should be within their scope of practice.

Participants in this study indicated that increasing patients’ awareness of the bidirectional association between diabetes and oral health may improve their acceptance and uptake of screening in the oral healthcare setting. Discussions with patients about increasing their awareness about T2D could be challenging for the OHP, particularly because patients may perceive this as being outside their area of expertise. The challenge is how to make the oral healthcare settings part of the identification of chronic disease and increase the awareness of the two-way relationship between chronic diseases and oral health [28].

Traditionally if an OHP suspected a patient may have diabetes a suggestion was made to “go and see your GP”, rather than a formal referral given to the patients about the need to explore their T2D status. OHPs have also reported that the practicalities of what can be done at the dental clinic depended on how things are progressed in the practice on that day [29]. Therefore, a referral pathway and model of care that provides a mechanism to facilitate referrals must be developed and maintained.

Currently a random capillary glucose (finger prick test) is used to monitor blood glucose in a patient diagnosed with diabetes, but this is not recommended to be used as a diagnostic test [30]. However, the use of accurate blood glucose monitoring technologies that do not require finger pricking (e.g., wearable technologies and bio-peripherals), will only increase in the future. Also, risk calculators can be implemented in mobile phones to identify high risk patients for delivery of targeted education (e.g., SMS-based information) and raising awareness for referral purposes. Still despite future advances, for the successful implementation of a screening programme in oral healthcare settings, several barriers need to be addressed. This will not happen without the concerted efforts of all healthcare professionals stakeholders and the adoption of new technologies and supporting infrastructure. It is also important to include innovations in healthcare delivery models which includes provider remuneration systems, and payment approaches [31].

Results for this study indicate that, although OHPs view screening for health conditions as part of their responsibilities, they do not normally conduct these screenings. It was also noted that the task of conducting screenings would not be compatible with OHPs operating in isolation. For screening in dental settings to be successful, the OHPs need to collaborate with GPs who would diagnose, provide follow-up and be responsible for the medical management of the patient. Participants commented that, to maximise the effectiveness of this approach to screening, improved referral pathways needed to be developed and more formal, co-ordinated, two-way communication and collaboration is essential between oral health professionals and GPs, as well as with diabetes educators, and nutritionists.

Additionally, participants mentioned several barriers to implementing diabetes screening, including resources such as time and the reimbursement of costs involved. OHPs also recognised that they may not have the required knowledge and training to deliver a diabetes screening protocol and manage patients following a risk assessment. These barriers need to be overcome to ensure individuals at risk of prediabetes and T2D are identified, enabling optimal management of their oral health and general health. This approach may also be applications to other general chronic conditions that can be screened for in the oral healthcare setting.

## Data Availability

The datasets generated and/or analysed during the current study are not publicly available due to the ethics approval granted on the basis that only researchers involved in the study could access the de-identified data. The minimum retention period is 5 years from publication. Supporting documents are available upon request to the corresponding author.

## Abbreviations

CPD: Continuing Professional Development
T2D: Type 2 diabetes
OHP: Oral healthcare professionals
GP: General Practitioner
